# Supplementary material for: Are carbon emissions trading and green financial instruments synergistic? -Comprehensive quantitative research based on content analysis
Source: PLoS One. 2024 Mar 7;19(3):e0298601. doi: 10.1371/journal.pone.0298601 (PMC10919601; doi:10.1371/journal.pone.0298601)
Supplement: S1 Data — (ZIP) [file pone.0298601.s001.zip › Data/地级市Z_F工作报告.zip!/╔╜╬≈/╜·│╟╩╨/└┤╫╘╣1⁄2╓┌║┼í╛┬φ┐╦╩2╛▌═°í┐_2007.docx]

各位代表：

　　现在，我代表市人民政府向大会作政府工作报告，请予审议，并请各位政协委员和其他列席人员提出意见。

　　一、2006年政府工作回顾

　　过去的一年，在市委的正确领导和市人大、市政协的监督支持下，市政府坚持以邓小平理论和“三个代表”重要思想为指导，按照科学发展观和构建社会主义和谐社会战略思想的要求，开拓进取，真抓实干，全市经济社会发展取得新的进步。

　　──国民经济较快增长。全年地区生产总值完成364.4亿元，增长11.8%；规模以上工业增加值完成179.4亿元，增长15.1%；粮食总产85.3万吨，增长7.9%，创建市以来最好水平；财政总收入（包括采矿权价款收入市县留成部分）完成103.49亿元，收入总量排全省第二，增长幅度为67.8%，比全省平均增速高29.5个百分点，排全省第一。剔除采矿权价款收入，正常财政总收入完成79亿元，排全省第四，同比增长28.1%，比全省平均增速高7.8个百分点。一般预算收入完成51亿元，剔除采矿权价款收入，正常一般预算收入完成26.5亿元，同比增长32.2%，比全省平均增速高10.8个百分点。财政收入占生产总值的比重达到21.7%，比全省平均水平高3个百分点，表明我市的经济增长质量越来越好；全社会固定资产投资完成164.7亿元，增长32.7%；社会消费品零售总额完成87.6亿元，增长16.1%。

　　──社会事业全面发展。全国文明城市、国家生态园林城市、国家环保模范城市、全国绿化模范城市和国家卫生城市创建活动顺利推进，科技、教育、文化、卫生事业得到加强。城镇登记失业率为2.4%，低于省控目标1.6个百分点。城镇基本社会保障覆盖率达到65%。

　　──资源环境治理保护工作得到加强。在经济保持较快增长的同时，综合能耗和污染物排放总量出现下降。全年万元地区生产总值综合能耗减少0.06吨标准煤，下降2.5%;万元地区生产总值主要污染物排放量减少17公斤，下降17.5%;二氧化硫和化学需氧量分别减排0.3万吨和0.1万吨，分别下降2.6%和4.8%；市区空气质量二级以上天数达到293天，比上年增加35天，位居全省第一。

　　──人民生活水平稳步提高。城镇居民人均可支配收入完成10132元，增长13.7%，增速比全省平均水平高出1.2个百分点；农民人均纯收入完成3939元，增长9.6%，绝对值比全省平均水平高出758元；城乡居民人均住宅面积分别达到30.1和25.6平方米，分别增长6.2%和2%。

　　这里，我向大会郑重报告，经过全市人民的共同努力，市五届人大一次会议通过的新的地区经济社会发展指标基本完成！为城乡居民办的20件实事按进度要求全部兑现！全市“十一五”发展实现良好开局！

　　一年来，我们主要抓了以下七个方面的工作：

　　1、扎实推进产业结构调整。以建设全国重要的煤化工基地为突破口，紧紧围绕“四大基地”、“八大产业”，积极实施项目带动战略，狠抓100个重点煤炭企业和100个重点非煤企业的“双百工程”，产业结构调整取得新的进展。煤炭资源整合有序开展，产业集中度有效提高。采矿权价款征缴完成56.8亿元。淘汰关闭矿井169个，关闭硫铁矿168个。王坡150万吨矿井正式投产，唐安150万吨扩建矿井竣工验收，大宁400万吨新建矿井和大阳150万吨扩建矿井具备竣工验收条件，伯方180万吨扩建矿井和望云西区60万吨新建矿井开始试产，东峰、伏岩山、苏村、曲堤、小窑沟等骨干矿井建设进展顺利，大宁金海300万吨矿井和兰花玉溪240万吨矿井开始前期工作。电力产业呈现稳步发展态势。阳城电厂二期工程进展顺利，晋城500千伏变电站全面投运，途经我市的晋东南—南阳—荆门百万伏级特高压输电试验示范工程开工建设，国投华实晋城热电联供项目和鲁能（沁水）电厂项目正在抓紧前期工作。煤化工基地建设方兴未艾。兰花阳化“813”项目、天脊中化“4060”项目和永丰“1830”项目建成投产，兰花田悦大颗粒尿素、晋城煤化工大颗粒尿素二期、天泽煤化工煤气化厂大颗粒尿素搬迁改造、兰花集团二甲醚一期、晋煤集团煤基合成油等一批煤化工项目开工建设。全市已经形成了260万吨大颗粒尿素、38万吨甲醇的生产能力。新兴产业和高新技术产业迈出了新的步伐。富士康科技工业园项目推进顺利，B区首期工程已经完成并开始生产，A区前期工作也已启动。煤层气开发利用步伐加快，抽采井建设、输送管道建设和用户市场开拓全面提速，全国煤矿瓦斯治理和利用工作现场会在我市召开。阳城建瓷园区新建生产线2条，新增年生产能力1200万平方米。旅游业注重资源整合和产品促销，呈现良好发展态势。王莽岭景区一期工程竣工开放，二期工程开工建设，申报4A级景区通过省级初评，为我市旅游业增添了新的亮点。全市全年接待游客652万人次，完成旅游总收入34.1亿元，同比分别增长17.7%和68.5%。民营经济快速发展，全年完成增加值141.8亿元，上缴税金20亿元，同比分别增长26.9%和27.5%。

　　2、扎实推进社会主义新农村建设。贯彻落实中央关于社会主义新农村建设的一系列方针政策，加大了对“三农”的支持力度，全年市、县两级财政安排农村各项资金5.23亿元，同比增长39.4%。认真兑现对农民种粮、购买农机具、发展畜牧业的补贴政策，农民的生产积极性进一步提高。出台了“十一五”时期社会主义新农村建设总体规划，制定了实施意见和配套措施，全面启动了新农村建设工作。编制完成建制镇总体规划19个、集镇（乡）总体规划7个、村庄建设规划150个，完成率分别达到85%、38%和24%。建成新农村示范村68个、达标村65个，移民搬迁自然村115个。农业结构调整步伐继续加快，粮食生产稳定增长，畜牧、蚕桑等特色农业加快发展，猪、牛、羊出栏分别增长13.6%、14.7%和1.6%，蚕茧总产量和总收入分别增长14%和62.9%，阳城蚕丝成为“国家地理标志保护产品”。农业产业化稳步推进，龙头企业完成产值32亿元，带动基地42万亩、农户20万户。江苏雨润集团200万头生猪屠宰加工冷鲜肉项目落户高平，引进战略投资者发展农业产业化迈出新的步伐。新发展农民专业合作经济组织80多个，提高了农民走向市场的组织化程度。乡镇企业保持较快增长，全年增加值、营业收入、提供税金分别增长23%、23.2%和25.8%。农业和农村基础设施建设取得新的进展。建成高标准旱作农田7.8万亩，完成土地开发整理复垦2.8万亩，新增耕地1.3万亩。完成农业节水灌溉4万亩，水保初步治理16万亩。加大了农机新技术的推广力度，机械化保护性耕作达到5.6万亩。完成造林24.9万亩，其中省级六大造林绿化工程6.4万亩。建成农村饮水安全工程147处，解决了300个自然村10.2万人的饮水安全问题。农村新能源建设走在全省前列，建成5512个户用沼气池、2个大中型沼气工程和10个秸秆气化站。新建村通水泥（油）路2074公里，改建县乡公路44.4公里，新增农村客运线路31条。

　　3、扎实推进城市基础设施和重点工程建设。以加快城镇化步伐为目标，以扩容提质为核心，加大了中心城市、4个县城和重点小城镇的规划、建设、管理力度。特别是围绕全国文明城市、国家生态园林城市、国家环保模范城市、全国绿化模范城市和国家卫生城市创建活动，狠抓了中心城市的基础设施建设和环境整治。赵树理文学馆建成开放，开发区供水分厂、旧城供水管网改造工程和市污水处理厂一期工程投入运行，城市生活垃圾无害化处理工程进展顺利，红星东街东延伸段、新市东街东延伸段和景西路道路工程建成通车，白水街开始路基施工。继续加大城市绿化力度，开展了“市树、市花”评选活动，新增生态绿地5876亩，建成5个绿化达标小区。中心城市建成区绿化覆盖率、绿地率和人均公共绿地面积分别达到45.3%、43.2%和15.5平方米，比上年分别提高了5.7个百分点、6.2个百分点和3平方米。“蓝天碧水工程”取得新的成效。市区空气综合污染指数控制在2.79，比上年下降0.8。“两河”综合治理工程荣获“中国人居环境范例奖”。市区饮用水源地水质达标率达到100%。与此同时，关系全市经济社会发展后劲的基础设施建设进一步加强。张峰水库枢纽工程完成年度投资1.46亿元，上、下游堆石和心墙堆石均已填筑到727米高程，一期移民工作基本结束；晋济高速公路工程完成年度投资7.96亿元，特大桥和大桥桩基、涵洞、通道已全部完成；阳城至关门高速公路前期工作基本结束，不久即可正式开工。

　　4、扎实推进改革开放工作。按照省委、省政府把晋城建设成为山西改革开放桥头堡的要求，积极深化改革，全力扩大开放，有力地促进了全市经济社会的健康发展。国有企业改革取得新的进展。制定出台了关于市属国有企业改革的13个配套文件，有88个市属国有企业完成了清产核资集中联审认定工作，晋城钢铁有限责任公司和阳城制丝有限责任公司破产工作正式进入法律程序。分离企业办社会工作推进顺利，中央驻市企业分离移交学校工作全面完成，省属、市属企业分离移交学校工作正在进行。乡村集体企业改革迈出新的步伐，有554个非煤企业进行了多种形式的改制，占总数的82.8%。煤炭企业改革开始起步，有359座煤矿完成了清产核资和资产评估，占总数的85.5%；有332座煤矿完成了产权界定，占总数的79%。大公司、大集团战略取得质的突破，兰花科创进入“最具价值上市公司”50强并再次募集资金4.3亿元，山西太行无烟煤发展集团挂牌运行并在进军煤化工产业方面迈出了实质性步伐，天泽煤化工公司成功与多家知名企业实现了战略合作。认真落实全省对外开放工作会议精神，加大了招商引资力度。通过广泛征集、认真筛选，开列了近300个项目的招商“菜单”。举办了首届海外留学人才晋城创业项目洽谈会，签订贸易、投资合同和技术合作协议60余项。组团参加了省政府组织的上海、香港招商活动和中部投资贸易博览会，签约项目61个，合同和协议引进资金383亿元。全年新批外资企业19家，合同直接利用外资额2.1亿美元。完成出口4973万美元，同比增长8.6%。开发区经济保持稳步发展，生产总值、工业增加值、财政总收入分别增长33%、25%和24.8%。

　　5、扎实推进资源节约和环境保护工作。狠抓节能降耗，制定了化工、煤炭、电力、冶铸等重点行业节能降耗考核指标，建立了县（市、区）单位生产总值能耗指标公报制度。对50户重点耗能企业实施了跟踪监督，对新上项目强化了能耗评价。加强了节能宣传，在媒体开辟了节能专栏，组织了节能宣传周、节能知识竞赛等宣传活动。加大了对煤矿废水、煤层气、焦炉尾气、粉煤灰和煤矸石等资源的综合利用力度，特别是煤层气开发利用走在了全国前列，煤层气地面抽采能力达到每年3亿立方米以上，煤层气发电厂装机容量达到4.3万千瓦，煤层气汽车达到1500多辆，民用煤层气用户达到2.6万多户。加大了对燃煤污染和工业粉尘污染的监管治理力度，对市区及周边12个企业实施了限产和关停。大力实施农村沼气工程、秸秆气化工程和小水电代燃料工程，全市近2万户农民告别了燃煤的历史，改善了农民生活环境和农村生态环境。污水处理设施建设进展顺利，巴公工业园区污水处理厂一期工程投入运行，晋煤集团生活污水处理厂主体已经建成，高平市生活污水处理厂开工建设。加大了生态示范区建设力度，陵川、沁水两个国家级生态示范区通过国家环保总局验收，创建国家级环境优美乡镇1个、省级环境优美乡镇3个、生态文明村13个。

　　6、扎实推进和谐晋城建设。在加快经济发展的同时，坚持以人为本，注重发展社会事业，高度重视解决民生问题。切实加强了就业和社会保障工作。全市新增城镇就业岗位4.18万个，为年度目标的2.1倍；输出和就地转移农村富余劳动力9.76万人，为年度目标的1.4倍；下岗失业人员再就业7764人，为年度目标的1.8倍，其中“4050”人员再就业1767人，为年度目标的2.1倍。加大了保险基金征缴力度，离退休人员养老金和并轨后“4555”、“4050”人员基本生活费做到了按时足额发放。城乡低保稳步推进，城市和农村受保障人数分别达到3.2万人和4.8万人。大力实施科教兴市战略，科技对经济社会发展的贡献率进一步提高。积极实施人才强市战略，全年引进副高以上职称和硕士以上学历人才40多人。义务教育阶段“两免一补”政策落实到位，完成标准化操场建设65个，改造农村中小学危房7.4万平方米，市区9所中小学新建扩建项目进展顺利，晋城学院开始筹建。组织开展了人民作家赵树理诞辰100周年纪念活动，成功举办了山西省第五届广场文化艺术节。公共卫生体系继续完善，突发公共卫生事件预防和处置能力进一步加强。推进和完善新型农村合作医疗制度，农民参合人数达到125万人，参合率达到88.3%。完成了40个乡镇卫生院标准化建设，农村卫生机构达标率达到60.8%。体育事业健康发展，参加第十二届省运会获得金牌数比上届实现翻番，承办了全国门球冠军赛山西赛区选拔赛和山西省第十二届运动会柔道项目赛，举办了全市第三届农民运动会。严格责任制落实和责任追究，安全生产实现第五个平稳年目标。“双拥”和“双服务”活动成效显著，国防教育和民兵预备役建设进一步加强。广播电视、民族宗教、妇女儿童、档案史志、外事侨务、文物保护、人民防空、气象、统计、地震、老龄、残疾人等各项工作都取得了新的成绩。

　　7、扎实推进民主法制和精神文明建设。市政府认真执行市人大及其常委会的各项决议，及时向市人大及其常委会报告工作。积极支持市政协参政议政，认真吸收政协常委会专题报告的建议，注重听取各民主党派、无党派人士和专家学者的意见，努力拓宽同人民群众的联系渠道。全年办理人大代表建议364件、政协委员提案275件，做到了事事有回音，件件有着落。对涉及人民群众切身利益的重大事项和重要决策通过公示、听证等多种形式，主动征询人民群众和社会各界的意见，推进了政府决策的科学化、民主化。高度重视政府法制工作，认真推行行政执法责任制，进一步规范了行政行为。积极推进基层民主政治建设，政务公开、企务公开和村务公开不断完善。“五五”普法全面启动，全民法制意识进一步增强。强化社会治安综合治理，深入开展严打整治专项斗争，积极化解各种矛盾，维护了社会大局稳定。认真落实政府系统党风廉政建设责任制，切实加强了行政监察和审计工作。认真贯彻全省政府系统干部大会精神，切实加强行政效能建设，严肃查处了行政效能案件21起，对30人进行了责任追究。广泛开展爱国主义和集体主义教育，大力推进以社会主义荣辱观教育为主的公民道德建设，继续深化“做文明晋城人，建文明晋城市”群众性精神文明创建活动，全国文明城市创建工作顺利推进。

　　各位代表，过去一年所取得的成绩，是贯彻执行党中央、国务院和省委、省政府一系列方针政策的结果，是中共晋城市委正确领导和市人大、市政协监督支持的结果，是全市人民同心同德、奋力拼搏的结果。在这里，我代表市人民政府，向全市人民，向驻市解放军指战员、武警官兵和公安干警，向各民主党派、工商联、各人民团体和社会各界人士，向所有关心和支持晋城发展的同志们、朋友们，表示衷心的感谢和崇高的敬意！

　　在肯定成绩的同时，我们也清醒地看到，前进中还存在不少矛盾和问题。主要是：产业单一、产品初级的资源型经济特征还比较突出，结构性矛盾还没有从根本上得到解决；经济增长方式还比较粗放，节能降耗和环境保护的压力较大；自主创新能力不足，技术进步还远不能成为经济发展的主要动力；制约农村经济发展和社会主义新农村建设的一些深层次矛盾还没有得到有效化解，保持农民收入持续增长还缺乏稳固的基础；发展面临的体制性、机制性矛盾仍然突出，还需在深化改革、扩大开放上狠下功夫；经济与社会发展还不够协调，促进和谐发展的任务还十分艰巨；政府职能转变和行政过错责任追究还没有完全到位，经济发展的软环境还不够宽松。这些困难和问题，有的是长期积累下来的，有的是体制转轨和结构调整过程中难以完全避免的，有的是工作不到位造成的，都需要我们采取有效措施，认真加以解决。

　　特别需要报告的是，一年来，尽管我们在节能降耗方面采取了一系列措施，单位地区生产总值能耗比上年有所下降，但未能完成市政府在市人大五届一次会议上提出的预期目标。尽管我们坚持不懈地狠抓安全生产，圆满实现了第五个安全生产平稳年目标，煤炭生产百万吨死亡率为0.35，比全国平均水平2.04低1.69，比全省平均水平0.87低0.52，但在安全生产方面还存在不少隐患，城区苗匠联办煤矿今年发生的“3·18”特大瓦斯事故就再一次给我们敲响了警钟。今年，我们一定要采取更加切实有效的措施，务求把节能降耗和安全生产工作做得更好。

　　二、2007年政府工作的总体要求和主要任务

　　2007年是深入贯彻科学发展观、推进和谐社会建设的重要一年，也是落实市第五次党代会精神的关键之年。做好今年的政府工作，对于保持全市经济社会又好又快发展，迎接党的十七大胜利召开，意义十分重大。今年政府工作的总体要求是：以邓小平理论和“三个代表”重要思想为指导，全面落实科学发展观，加快构建社会主义和谐社会，在调整产业结构、转变增长方式上求突破，在推进资源节约、加强环境保护上求突破，在统筹城乡发展、推进社会主义新农村建设上求突破，在加强城市基础设施建设、提升城市管理水平上求突破，在继续深化改革、进一步扩大开放上求突破，在发展社会事业、解决民生问题上求突破，努力实现经济社会又好又快发展，以优异成绩迎接党的十七大胜利召开。

　　按照新的地区经济社会发展指标体系，综合考虑各种因素，今年全市经济社会发展的主要预期目标是：地区生产总值增长12%，固定资产投资增长28%，规模以上工业增加值增长16%，财政总收入、一般预算收入分别增长16%和18%（均不含采矿权价款收入），单位地区生产总值综合能耗完成省定目标，二氧化硫、化学需氧量减排分别下降0.9%和4%，市区空气综合污染指数控制在2.6以下，空气质量二级以上天数达到295天以上，城镇登记失业率控制在4%以内，城镇居民人均可支配收入增长12%，农民人均纯收入增长8%，社会消费品零售总额增长15%，居民消费价格总水平涨幅控制在3%以内，外贸进出口总额增长22%，人口自然增长率控制在6‰以内。

　　做好今年的政府工作，要把思想和行动统一到中央和省委、省政府对当前发展环境的分析和判断上来，统一到中央和省委、省政府对贯彻落实科学发展观的要求上来，统一到中央和省委、省政府对经济社会工作的决策和部署上来。要深刻认识市情特点和发展的阶段性特征，正确处理“好”与“快”的辨证关系，在“好”的基础上能快则快。要正确把握当前面临的发展机遇和严峻挑战，既要看到发展条件进一步改善，发展环境进一步优化，发展势头进一步趋好，又要充分正视影响经济社会发展全局的突出问题，充分正视长期积累的体制机制性矛盾，充分正视发展中出现的新情况新问题，保持清醒头脑，抓住发展机遇，更加积极主动地做好工作。要把又好又快发展作为贯彻落实科学发展观的本质要求，努力实现速度、质量、效益，消费、投资、出口，人口、资源、环境三个方面相协调，保持经济平稳较快发展，推动经济社会切实转入科学发展的轨道。

　　为圆满完成今年经济社会发展的奋斗目标，实现好中求快、又好又快发展的战略意图，我们要扎扎实实抓好以下工作。

　　1、以加快建设煤化工基地为切入点，努力调整优化产业结构

　　紧紧抓住国家鼓励东部地区向中西部地区进行产业转移的有利时机，继续以建设全国重要的煤化工基地为抓手，以“四大基地”、“八大产业”为重点，以“产业园区和产业带”为载体，以“双百工程”为支撑，加快产业结构调整步伐。

　　继续提高煤炭产业集中度和产业素质。我市调整产业结构必须首先做好煤炭这篇大文章，着力改变以严重破坏生态环境、过度浪费资源为代价的发展模式，推动煤炭工业走集约发展、内涵发展、绿色发展、高效发展和安全发展的道路。要以国家在我省开展煤炭工业可持续发展政策试点为契机，打好打胜省政府组织实施的煤炭产业“三大战役”，提高煤炭产业集中度和产业素质，增强市场竞争力和调控力。继续坚持“关小、改中、建大”的原则，抓紧完成煤炭资源整合和有偿使用工作，确保第三批整顿关闭矿井不折不扣落实到位。推进年产30万吨以下矿井的二次资源整合，淘汰落后生产能力。为确保实现保护资源和安全生产的目的，今后关闭矿井要改变以往简单地把指标按比例分配到县（市、区）的做法，全市统筹考虑，严格按照矿井生产规模和安全生产标准确定是否关闭。继续加快骨干矿井建设，今年大宁、大阳、唐安、伯方等骨干矿井要正式竣工投产，武甲、小西和坪上等矿井要开工建设。全市地方煤矿采掘机械化程度要达到35%、煤炭资源采区回采率要达到65%、洗选率和加工转化率要分别达到30%、块炭率要达到40%。继续建设完善瓦斯监控、产量监控和井下人员考勤定位监控三大系统，建成本质安全型矿井1座，安全高效型矿井10座，30万吨以上单独保留矿井全部达到质量标准化要求。

　　积极发展以煤化工为重点的新兴优势产业。经过近几年的努力，我市煤化工产业已经形成了强劲的发展势头。去年国家发改委规划出台了全国七大煤化工基地，我市位列其中。我们要紧紧抓住当前的有利时机，加快煤化工产业发展步伐。今年，要重点抓好已投产项目的达产达效，抓好兰花田悦大颗粒尿素、晋城煤化工大颗粒尿素二期、天泽煤化工煤气化厂大颗粒尿素搬迁改造、兰花集团二甲醚一期、晋煤集团煤基合成油等已开工项目的建设进度，抓好晋丰化工“3652”二期、兰花集团10万吨聚甲醛和60万吨聚丙烯、欣荣公司60万吨甲醇20万吨二甲醚、皇城集团40万吨甲醇10万吨二甲醚、太行无烟煤100万吨二甲醚、丹丰化工30万吨甲醇二期等项目的前期工作。电力产业要开工建设兴高焦化废气余热发电项目，力争开工建设国投华实晋城热电联供项目。冶铸产业要积极发展精密铸造和零部件加工，重点抓好福盛钢厂200万吨钢材、山西汉通100万件汽车零部件等项目建设。高新技术产业要加快富士康晋城科技工业园建设，今年要全面建成B区项目，开工建设A区项目。煤层气产业要在继续增加抽采能力的同时，加快煤层气液化项目和管道集输项目建设，积极拓展用户市场。新型建材业要以阳城建瓷工业园区为龙头，积极推进产品结构由单一、低档向多元、中高档转变。农副产品加工业要以特色农业基地为依托，壮大厦普赛尔饮品、白马王啤酒、鸿生淀粉、塔王醋业等龙头企业，加快建设江苏雨润集团200万头生猪屠宰加工冷鲜肉项目。

　　全力壮大旅游产业。继续完善服务功能，改善旅游环境，做好中国优秀旅游城市复核工作。注重整合旅游资源，突出皇城相府和王莽岭两大景区的龙头地位，抓好沁河旅游区、城郊古建景区和陵川礼义宋金古建景区建设，打造“神奇太行·经典晋城”的旅游品牌形象。加强重点文物的科技防范和修缮维护工作，充分发挥好文物资源的旅游功能，实现文物保护和利用相统一。继续鼓励社会投资者按照政府统一规划，采取特许经营模式，开发建设旅游景区。鼓励外地有实力的旅行社来我市设立分支机构，提升我市旅游业服务水平。加大旅游促销力度，推动旅游业快速发展。

　　加快发展服务业。积极运用新型商业业态、现代经营方式和服务技术改造提升商贸流通业。推进城市商业网点建设，积极发展专业连锁超市，规范发展小商品市场，完善社区商业服务功能。继续推进“万村千乡”试点工程，加快“农家店”改造和建设步伐，构建集中采购、统一配送的农村商业流通网络。积极提升餐饮、住宿、房地产等传统服务业，大力发展证券、中介、信息咨询、文化娱乐、体育、社区服务等现代服务业，努力提高服务业占生产总值的比重。特别是要重视发展金融服务业，充分利用基金、企业债券等现代金融工具，拓展企业融资渠道。

　　加强基础设施建设。今年，要集中力量抓好关系全局的17项重点工程。其中，续建10项，分别为张峰水库、阳电二期、晋济高速公路、城市生活垃圾无害化处理工程、晋城矿区采煤沉陷区综合治理工程、晋城二中教学楼、兰花集团二甲醚项目、富士康晋城科技工业园、晋煤集团长平煤矿和晋煤集团棚户区改造项目；新开工7项，分别是阳城至关门高速公路、嘉南铁路、端氏220千伏输变电工程、市区东南出入口通道工程、晋城煤层气综合开发利用项目、晋城学院和市委党校。

　　各位代表，调整优化产业结构，必须进一步扎扎实实做好项目工作。全市上下要按照省政府提出的项目攻坚年和建设年的要求，加大项目工作力度，促成一批重大产业项目引进建设和投产达效，同时要十分重视新上一批中小项目，切实做到大项目“顶天立地”，中小项目“铺天盖地”，形成以项目支撑又好又快发展的良好格局。

　　2、以增加农民收入为切入点，努力推进社会主义新农村建设

　　扎实推进社会主义新农村建设。坚持工业反哺农业、城市支持农村和“多予少取放活”的方针，按照“生产发展、生活宽裕、乡风文明、村容整洁、管理民主”的要求，加强组织领导，完善建设规划，坚持从实际出发，因地制宜，分类指导，积极探索不同类型地区的发展模式，在巩固提高去年新农村建设成果的基础上，再启动100个新农村试点示范村，建设100个新农村达标村，搬迁100个自然村，使我市新农村建设走在全省前列。

　　积极探索“以工补农”、“以城带乡”的体制机制。完善财政支农资金的稳定增长机制，切实加大对“三农”的投入。继续落实对农业和农民的直接补贴政策，支持县级政府将上级下达的支农资金捆绑使用，提高支农资金使用效益。完善以工补农、以城带乡的社会参与机制，推动城市交通、物流、商贸和公共服务业向农村延伸，继续开展党政机关、事业单位扶贫帮困和支持新农村建设活动，提倡效益好的工商企业按照“一企一村”的模式对口帮扶企业所在地或相邻地区的农村。积极探索适应社会主义市场经济的农村集体经济有效实现形式，逐步解决目前一些农村集体经济成为“空壳”甚至债台高筑的问题，增强基层组织服务“三农”的能力。以项目落地为抓手，大力推进陵川县“两区”开发建设。

　　加快发展现代农业。进一步加快农业结构调整步伐，抓好畜牧、蚕桑、蔬菜、小杂粮、干鲜果、中药材等特色基地建设，努力发展绿色高效优质农业，提高农业比较效益。按照工业化的思路，着力发展农业产业化经营，推进生产、加工、储运和销售一体化，培育壮大一批龙头企业。完善农业基础设施，改善农业技术装备，努力提高农业水利化、机械化和信息化水平，增强农业抗灾防灾和综合生产能力。加强农业科技推广和农民素质培训工作，实施好“百万农民素质提高工程”、“农业科技入户培训工程”和“农村劳动力转移阳光工程”。抓好农民专业合作经济组织和农村行业协会的发展与规范工作，提高农民走向市场的组织化程度，以农业组织化促进现代农业发展。发挥小城镇的集聚和带动效应，大力发展农村二、三产业，发展和壮大县域经济，推进农村现代化，转移农民，减少农民，富裕农民。

　　大力推进沼气和秸秆气“户户通”工程。发展农村沼气和秸秆气是集经济效益、生态效益和社会效益为一体的民心工程，对于改善农民生活环境和农村生态环境，推进社会主义新农村建设，具有重大意义。要紧紧抓住国家实施农村新能源建设，以及国家农业部拟把我市作为生态家园示范市的大好机遇，统一认识，统一行动，集中人力、物力、财力，大打一场沼气和秸秆气“户户通”攻坚战。今年，全市要新建农村户用沼气池6.5万个、大中型沼气工程50个、秸秆气化站50个，使10万农户用上新型清洁能源。到2010年，全市有条件的地区要实现沼气和秸秆气“户户通”，30万农户用上新型清洁能源。对沼气和秸秆气工程建设，市、县两级财政要给予重点扶持。同时，积极发展煤层气和小水电代燃料等清洁能源。

　　加快建设“四供八库”新水源工程。合理开发利用水资源，事关经济社会发展大局。目前，我市人均水资源占有量为626立方米，不论在山西来讲，还是在周边地区来讲，都属于相对富水区。但由于缺少地表骨干水源控制工程，生产生活取水以地下水为主，导致地下水严重超采，而每年有近11亿立方米的地表水资源白白流向境外，我市又是一个典型的工程型缺水区。随着经济社会的快速发展，水的供需矛盾将更加突出，预计到2010年全市年需水量将达到5.2亿立方米，需要新增供水能力2.8亿立方米。因此，建设新的水源工程，已成为保障全市经济社会又好又快发展的战略任务。从今年开始，我们要按照省委、省政府实施兴水战略的工作部署，加快建设“四供八库”新水源工程，破解水“瓶颈”制约，满足全市经济社会发展用水需求。“四供”即下河泉供水工程、任庄水库供水工程、固县河供水工程和丹河下游供水工程；“八库”即张峰水库、东焦河水库、围滩水库、西冶水库和水电电气化县项目的磨滩、曹河、湾则、秦家磨4座调蓄水库。目前，张峰水库正在紧张建设之中，东焦河水库今年即可下闸蓄水。今年，要力争开工建设其它项目。要以市场经济的思路和办法，积极探索政府规划引导、市场运作筹资、企业建设运营的水利建设新模式，引导社会资金投入水源工程建设，实现投资者和用水者的共赢。在全社会强化水商品意识，探索建立水资源保护和合理开发利用的体制机制，用经济手段限采地下水，抓好节约用水工作。加强原有水利工程的维修配套、挖潜增效，建设好人字闸、淤地坝以及旱井、水窖等雨水集蓄利用工程，抓好中小型水库的除险加固。继续实施农村饮水安全工程，解决10万农村人口饮水安全问题。

　　全力推进通道绿化和交通沿线荒山绿化。以创建全国绿化模范城市为抓手，按照“公路两侧树成荫、通道视线无荒山”的要求，全力推进以通道绿化和交通沿线荒山绿化为重点的六大造林绿化工程。重点是进一步做好长晋、晋阳、晋焦三条高速公路的补栽补植工作，完成晋焦高速公路出口绿化、196公里一二级国省道绿化、136公里宽林带景观通道绿化和1.4万亩交通沿线荒山绿化。对已经完成的通道和交通沿线荒山绿化，要加强管护，及时补植，确保绿化效果。

　　加快发展农村社会事业。加大对农村教育事业的投入力度，继续抓好农村中小学危房改造，完善提升100所农村寄宿制学校。按照社会主义新农村建设规划和移民并村要求，积极稳妥地做好农村中小学撤并工作，进一步优化中小学布局。统筹教师资源，加强农村学校师资队伍建设，逐步解决农村教师不足和整体水平不高的问题。完成农村远程教育网络建设。加强农村基层文化设施建设，推进文化先进县、文化先进乡（镇）和特色文化村“三级联创”工作，实施好农村广播电视“村村通”工程、文化信息资源共享工程和农村电影放映工程，丰富和满足农民群众的精神文化生活。健全县、乡、村三级医疗卫生服务体系，完善新型农村合作医疗制度。实施农村医疗救助制度，健全农村“五保户”供养、社会互助、灾民救助等社会救助体系。

　　各位代表，“三农”工作是政府工作的重中之重，我们一定要带着对农民群众的深厚感情，以建设社会主义新农村为抓手，千方百计地增加农民收入，筑牢农业基础，推进农村发展。

　　3、以改善人居环境为切入点，努力提高城市建设和管理水平

　　切实搞好城市规划。紧紧围绕“十一五”经济社会发展要求，按照高起点、大手笔、精品化、特色化的思路，提升城市规划编制水平，充分发挥城市规划对城市建设和管理的指导作用。完善城市总体规划，完成市域城镇体系规划，确定近期建设规划，编制北石店区分区规划、旧城区修建性详细规划、建成区居民贴身绿地规划和景西路城市设计。完善城市规划管理制度，严格规划审批程序。

　　精心实施城市建设重点工程。一是“蓝天工程”。通过关停搬迁改造市区及周边污染物排放量大的工业企业、扩大集中供热供气、实施生活垃圾无害化处理、控制扬尘污染、加大机动车尾气治理等措施，确保市区空气质量继续改善。在实施“蓝天工程”中，要突出抓好集中供热、供气工程建设，今年要完成集中供热四期扩容工程和五期新建一期工程，市区煤层气综合开发利用项目年底要具备供气条件；二是“畅通工程”。开工建设市区东南出入口通道工程和前进路新市西街至凤台西街段道路工程，完成市区与北石店区连接线道路工程前期工作。开工建设汽车客运东站；三是“绿化工程”。完成城市东出口景观绿化工程，启动泽州公园和植物园改造工程，建成5个绿化达标小区，抓好城市周边山体绿化，新增绿地面积1500亩，推行绿色图章制度，加快国家生态园林城市创建步伐；四是“安康居住工程”。完成经济适用住房建筑面积8.1万平方米，普通住房建筑面积29.4万平方米。在抓好以上重点城建项目的同时，要精心规划，统筹运作，以晋城学院和市委党校建设为起步，推进城市新区开发。

　　进一步提高城市管理水平。坚持建管并重，标本兼治，构建城市管理长效机制。强化属地管理，注重发挥街道和社区居委会作用，形成全方位、多层次、多系统的齐抓共管局面。推进城市环卫体制市场化改革，全面放开道路清扫、垃圾清运和公厕保洁市场，变“以钱养人”为“以钱养事”。加大市容环境整治力度，对乱搭、乱建、乱画等群众反映强烈的“三乱”问题，要出新招、出重拳加以整治。城市管理要进一步向背街小巷、“城中村”和城乡结合部延伸，消除管理盲区和盲点。引导广大市民增强城市意识，提高文明素质，树立文明形象，营造热爱晋城、关心晋城、建设晋城的社会氛围，全方位提高城市文明程度，推进全国文明城市创建工作。

　　进一步加大经营城市力度。继续加大土地收购储备力度，严格执行经营性用地“招、拍、挂”制度，公开拍卖户外广告发布权和城市道路、桥梁、广场、游园冠名权，制定出台城市供热价格改革办法及城市污水和垃圾处理费征收办法。按照先易后难、分步实施的原则，加快市政公用事业改革步伐，今年要完成市自来水公司、市污水处理厂的市场化改革和市煤气公司的委托经营，用向社会招商的办法建设集中供热五期工程。

　　4、以进一步解放思想、更新观念为切入点，努力加快改革开放步伐

　　加快推进企业改革。今年我们要继续作为“企业改革攻坚年”，全面加快改革步伐。国有企业要按照“发展壮大一批，转制搞活一批，关闭破产一批”的思路，加速推进改革进程。积极引导市属国有企业的联合、兼并和重组，推动国有资本向煤炭、化工、电力等优势行业集中，重点推动太行无烟煤集团规范运行，加快推进天泽煤化工公司上市的前期工作；对目前竞争力不强但有发展前景的国有企业，要采取存量出售和增量引进相结合的方式，实现民营化或股权结构多元化，增强发展活力；对产品无市场、长期亏损且扭亏无望、资不抵债，以及生产工艺落后、高耗能、高污染的企业，要坚决实施关闭或依法破产。晋钢和阳城制丝两家企业今年上半年要完成破产，凤凰织品、绿洲纺织、百纺公司等列入第一批改制重点的35户企业年内要力争完成改制任务。县、乡两级政府要立足长远利益，把企业改革切实抓在手上，确保国有和城乡集体企业改革到位，运行规范，活力增强。煤炭企业要在完成资源整合和有偿使用工作的基础上，同步推进产权制度改革。

　　大力发展非公有制经济。着力在“改”、“扩”、“创”上下功夫，推动非公有制经济加快发展。“改”，就是要加快城乡集体企业改制步伐，推进城乡集体企业民营化；“扩”，就是要鼓励支持非公有制企业扩大规模，提高效益；“创”，就是要营造发展环境，鼓励全民创业。同时，进一步放宽市场准入，鼓励非公有制经济参与国有企业改革，进入基础设施、公用事业、农业开发和社会事业等领域。引导非公有制企业依法经营，保护非公有制企业合法权益。

　　深化投融资体制、财政体制和国有资产监管体制改革。改善政府投资管理模式，建立健全政府重大投资项目的决策咨询和论证制度。强化企业投资主体地位，畅通社会投资渠道，特别是要鼓励、引导民间资本更多地投向生产领域。建立完善银企联席会议制度，搭建重点建设工程、重大产业项目和中小企业融资平台。引导驻市的国有商业银行、股份制银行和政策性银行，正确执行国家的宏观调控政策，优化贷款审批程序，更好地服务和支持地方经济发展。引导城市商业银行和农村信用合作社发挥政策灵活、贴近基层的优势，为中小企业发展和新农村建设做出积极贡献。支持信用担保体系建设，为中小企业融资提供有效服务。完善公共财政框架，优化财政支出结构。进一步深化部门预算、国库集中支付、政府采购和收支两条线改革。根据全省财政体制改革要求，做好财政体制调整工作。强化国有资产监管，建立健全国有资本经营预算制度和国有资产经营业绩考核体系，完善国有企业重大财务事项报告制度、重大投资决策失误和重大财产损失责任追究制度，落实国有资产增值保值责任，规范政府与企业的分配关系。

　　推进农村综合改革。坚持和完善农村土地承包制度，依法规范土地承包经营权流转程序和行为。改革和完善征地补偿制度，切实维护农民权益。抓好乡镇机构改革、农村义务教育改革和县乡财政管理体制改革，建立精干高效的农村行政管理体制、政府保障的农村义务教育体制和覆盖城乡的公共财政制度。启动集体林权制度改革，增强林业发展活力。

　　继续加大对外开放力度。抓住国内外产业加快梯度转移、资本加速流动的契机，充实完善招商引资项目库，创新招商方式，突出企业的主体作用，积极参加“中部贸易投资博览会”、“中国（太原）国际煤炭与能源新产业博览会”等大型招商贸易洽谈活动，提高招商引资的质量和水平。继续强化“好苹果先吃”的理念，舍得拿出优势项目和优质资产招商引资，吸引更多的大企业、大集团落户晋城，引进战略投资者，以存量带动增量。加快推进与中海油集团、中化集团等大型企业集团在煤化工产业方面的战略合作。继续强化“晋城之外都是外”的理念，项目不论来自哪里，只要符合产业政策，技术先进，市场看好，都可以大胆引进。进一步完善重点产业发展规划，加快产业园区和产业带基础设施建设，做好项目用地的置换和储备，为项目的引进和建设提供载体。注重优化出口产品结构，扩大铸管、轻纺产品和空调压缩机、汽车零部件的出口规模，培育农副产品和高新技术产品出口基地。立足我市的产业特色和区位优势，实施好“南下战略”，积极融入中原经济圈和城市群，参与区域经济分工和合作。

　　各位代表，改革滞后，开放不足，是制约我市经济社会发展的重要因素之一。我们必须按照省委、省政府把晋城建设成为山西改革开放桥头堡的要求，进一步解放思想，更新观念，深化改革，扩大开放，为又好又快发展注入新的活力。

　　5、以节约能源资源和保护生态环境为切入点，努力转变经济增长方式

　　要以争创国家环保模范城市为载体，以实现节能降耗和污染减排目标为重点，把强化政府责任作为关键环节，采取有力措施，充分挖掘潜力，努力扭转资源、环境压力过大的局面，真正把经济增长方式转移到集约增长的轨道上来。

　　大力发展循环经济。抓住山西进入全国循环经济试点省的有利时机，积极创建循环经济示范市，加快我市循环经济发展步伐。积极在产业层面构建生态产业链条，在企业层面推行清洁生产，重点在煤炭、电力、冶铸、建材、丝麻纺织等产业，按照循环经济的要求，积极改进设备、工艺和流程，培育一批循环经济试点示范企业。抓好煤炭等资源型初级产品的加工转化，抓好煤矿废水、煤层气、焦炉尾气、粉煤灰和煤矸石等资源的综合利用。巴公工业园区要力争建成全省循环经济示范园区，20户循环经济试点示范企业要尽快形成废弃物和副产品的循环利用产业链条。在抓好循环型工业和循环型园区的同时，要在循环型农业、循环型服务业、循环型社会等领域铺开试点，树立样板，带动全市循环经济加快发展。

　　抓好节能降耗工作。突出抓好煤炭、冶铸、焦化、化工、电力、建材等重点行业的节约资源和节能、节材工作，制定科学的耗能指标，建立健全计量管理制度、能源消费统计制度和节能工作奖罚制度，引导企业通过改进设备工艺、强化运行管理等有效措施节能降耗。今年，要对年消耗5000吨标准煤以上的50户高能耗企业实施重点监控。继续下大力气淘汰落后生产能力，引导、支持拟淘汰企业转产、技改或新上符合国家产业政策、符合节能和环保要求、技术起点较高的产业项目。推广兰花集团原材料集中采购和内部市场化的做法，重点企业单位产品原材料消耗要力争下降10%以上。大力推进城市节水工作，加大自备水源井关闭力度，推广节水型器具，推进中水循环利用。积极发展农业节水灌溉，加大小水电代燃料工程建设力度。发展节能、省地型住宅和公共建筑，推进建筑节材，新建民用住宅全部实行分户供暖。认真执行最严格的土地管理制度，切实加强耕地特别是基本农田保护，努力盘活存量土地，千方百计节约城乡建设用地，优先保障重点基础设施、重点产业项目和重点社会事业项目的用地需求。

　　坚持把生态环境保护放在突出位置。做好矿区沉陷区综合治理工作，加快矿区生态恢复，建设绿色生态型矿区。加快市区重点污染企业的关停搬迁治理步伐，严禁高硫劣质煤进入市区，严格控制市区废水、废气和固体污染物的排放，建成区烟尘控制区覆盖率达到90％以上。积极实施“碧水工程”，晋煤集团生活污水处理厂和高平市生活污水处理厂要建成使用，阳城县生活污水处理厂要开工建设，陵川县和沁水县生活污水处理厂要完成前期工作。同时，实施好丹河中下游植物净化工程，抓好丹河流域污染治理，确保丹河水质明显改善；加强沁河流域环境监管，确保沁河水质稳定良好。继续加强生态示范区建设和旅游沿线生态环境保护工作。

　　各位代表，作为一个典型的资源型城市，资源型产业为我市区域经济的发展做出了巨大贡献，一方水土养了一方人；反过来，我们也必须切实注重产业发展与资源节约和生态保护相协调，一方人也必须护好一方水土。这是各级政府的责任，也是全市人民的责任。我们必须进一步统一思想，坚定信心，攻坚克难，务求实效。

　　6、以促进经济社会相协调为切入点，努力推动社会事业加快发展

　　大力推进技术创新和科技进步。营造有利于技术创新和科技进步的政策环境和文化氛围，培养全社会技术创新和科技进步意识。引深质量兴市活动，扎实推进名牌战略，努力创建全国质量兴市先进市。深化市直科研机构改革，有效整合科技资源，科技人员要沉到企业去，蹲在项目上，围绕重点产业的关键技术开展引进、示范和推广工作。广泛建立企业与高校、科研机构及企业间的技术联盟，发挥好企业在技术创新中的主力军作用。认真组织实施一批重点科技项目，提高产品科技含量，增强产业竞争能力。通过合作攻关和引进吸收，尽快掌握煤化工、煤基合成油、煤层气开发等方面的关键技术，推进产品升级和产业延伸。充分重视人才在技术创新和科技进步中的重要作用，紧紧抓住培养、引进、使用三个环节，加强高层次人才和实用型、技能型人才队伍建设，为经济社会又好又快发展提供人才支撑。

　　坚持优先发展教育事业。继续合理调整教育结构，重视幼儿教育，提升义务教育，普及高中阶段教育，扩大职业教育，发展高等教育，促进各级各类教育持续、健康和均衡发展。更加注重教育的普惠性，积极促进教育公平，推动公共教育资源向农村和贫困地区倾斜，缩小城乡教育差距。落实好国家义务教育阶段“两免一补”政策，认真做好进城务工农民子女的义务教育工作。继续大力推进民办教育，增强教育事业发展活力。积极发展职业教育，建立健全覆盖城乡的职业教育和培训网络，为经济社会发展培养实用型和应用型人才。加大教育基础设施建设力度，抓好晋城二中、中原街中学、秀水苑学校等9所市区学校的新建、扩建工程。

　　加快发展文化、卫生、体育等社会事业。抓住我市被确定为全国文化体制改革试点地区的契机，积极推进文化体制改革，不断激活旅游文化、广播影视、新闻出版等领域的内在活力。大力发展文化事业和文化产业，完善社区和农村公共文化设施，扶持发展民办文化，着力打造一批文化精品，为人民群众提供形式多样的文化服务。做好非物质文化遗产的挖掘、整理、保护和传承工作，继续申报省级、国家级非物质文化遗产保护项目。推进国家卫生城市创建工作，确保完成创建目标。完善城乡医疗卫生服务体系、疾病预防控制体系、卫生执法监督体系和突发公共卫生事件应急体系，大力发展社区和农村卫生服务，加强医疗市场监管，全面推行药品和医疗器械竞价采购，努力解决群众看病难、看病贵问题。年内完成市传染病医院建设。高度重视人口和计划生育工作，完善流动人口计划生育综合管理服务机制，稳定低生育水平，提高出生人口素质。加强社区和农村体育设施建设，倡导全民健身运动，提升竞技体育水平。重视国防教育，完善国防动员机制，深入开展“双拥”和“双服务”活动，加强人防工作。继续做好广播电视、民族宗教、妇女儿童、档案史志、外事侨务、文物保护、气象、统计、地震、老龄、残疾人等各项工作。

　　7、以改善民生为切入点，努力解决好关系群众切身利益的重大问题

　　进一步做好扩大就业工作。按照省政府的安排部署，积极开展统筹城乡就业试点工作。进一步完善公共就业服务体系，注重发展就业容量大的劳动密集型产业、服务业和中小企业，拓宽城乡居民就业渠道。加强职业培训，提高劳动者的就业技能。重点做好下岗失业和破产关闭企业人员的再就业工作，积极帮助“零就业家庭”和就业困难人员就业，加强对高校毕业生的就业指导和服务，推进退役军人安置改革。今年，全市要新增城镇就业岗位2.1万个。

　　努力完善社会保障体系。扩大养老、失业、工伤和医疗保险的覆盖面，强化社会保险基金的征缴和监督管理，确保按时足额发放，严禁侵占挪用。继续巩固和完善城乡最低生活保障制度，高度重视城镇化进程中失地农民的保障问题。加快建立适合农民工特点的社会保障制度，重点推进农民工工伤保险和大病医疗保障工作。关注弱势群体，健全城乡社会帮扶救助体系，帮助特殊困难群体解决基本生活和子女上学、看病就医等方面的实际困难。鼓励发展慈善事业。建立廉租住房制度，帮助城市低收入家庭解决住房困难。

　　继续完善收入分配制度。提高劳动因素在分配中的比重，努力增加城乡居民收入，特别是中低收入者的收入。引导各类企业在提高效益的基础上增加职工工资，加强对企业落实最低工资制度的监督检查，健全防止拖欠农民工工资的长效机制，切实维护劳动者合法权益。统筹解决好企业离退休人员、城乡低保对象和各类优抚对象的福利待遇。清理规范并实施统一的公务员奖金和津贴补贴制度，改革事业单位分配制度。

　　切实加强公共安全管理。进一步建立健全公共安全事件预警体系和应急处理机制，全面提升应对各种自然灾害和突发事件的能力。牢固树立“以人为本、珍爱生命”的理念，切实把安全生产抓在手上，强化责任意识，注重综合治理，加大投入力度，完善监管体系，提高监管水平，建立长效机制，严格责任追究，依法从重从快打击非法违法行为，坚决遏制煤炭、特种设备、危险化学品、非煤矿山等行业以及交通、公共聚集场所等领域重特大事故的发生，努力保持安全生产的平稳态势。特别是要认真吸取城区苗匠联办煤矿“3·18”特大瓦斯事故的深刻教训，居安思危，警钟长鸣，坚决克服思想上麻痹、工作上自满、管理上松懈的倾向，始终保持如履薄冰、如临深渊的警觉和责任，关口前移，强化监管。对存在重大安全隐患而整改不力者，要比照发生事故严肃处理；对非法生产的矿井，要一律实施关闭；对非法组织生产者，要一律依法追究刑事责任；对失职渎职的监管人员，要一律从严追究党纪、政纪和法律责任。

　　各位代表，做好上述几项工作，对维护好、实现好人民群众的根本利益，推进和谐晋城建设，具有重要意义。我们一定要聚精会神，全力以赴，狠抓落实。

　　8、以加强社会主义民主法制和精神文明建设为切入点，努力营造经济社会发展的良好环境

　　加强社会主义民主政治建设。政府要主动向人大及其常委会报告工作，自觉接受人大及其常委会的法律监督，认真执行人大及其常委会的决定、决议，支持人大代表依法行使职权。加强与人民政协的协商，接受人民政协的民主监督。认真办理人大代表建议和政协委员提案。广泛听取各民主党派、工商联和无党派人士的意见，充分发挥工会、共青团、妇联等人民团体的桥梁和纽带作用，拓宽与人民群众的沟通渠道。加强基层民主政治建设，继续完善基层群众自治组织和企事业单位的民主管理制度，扩大政务、企务、村务公开的范围和内容，保障人民群众的知情权、参与权和监督权。完善政府和政府主要部门的新闻发言人制度，增加政府工作透明度，接受社会监督。

　　加快依法治市步伐。进一步落实行政执法责任制，强化行政执法监督，推进依法行政，规范行政行为，建设法治政府。深入开展“五五”普法教育，增强公民的法制观念和依法办事、依法维权意识。认真落实社会治安综合治理措施，推进科技强警全国示范城市建设，深入开展“严打”整治斗争，严厉打击 “法轮功”等邪教组织和境外敌对势力的渗透破坏活动，严厉打击严重刑事犯罪尤其是多发性侵财犯罪和严重暴力犯罪，严厉打击破坏社会主义市场经济秩序的违法犯罪。强化信访、行政复议和社会矛盾排查调处工作，畅通群众诉求渠道，努力把各种不安定因素化解在基层和萌芽状态，坚决控制赴省、进京非正常群体性越级上访。在征地拆迁和企业重组破产中注重依法行政，努力维护农民、城市居民和企业职工的合法权益。加大整顿和规范市场经济秩序力度，严厉打击各种假冒伪劣等商业欺诈行为，创造良好的消费环境。

　　加强社会主义精神文明建设。弘扬以爱国主义为核心的民族精神和以改革创新为核心的时代精神，深入实践社会主义荣辱观，大力促进以社会主义核心价值体系为根本的和谐文化建设。推进社会诚信体系建设，深化社会公德、职业道德和家庭美德教育，培养公民的高尚人格，建立和谐的人际关系。普及科学知识，反对封建迷信，严厉打击“黄赌毒”等丑恶行为，倡导文明健康的生活方式。以“做文明晋城人，建文明晋城市”为主题，广泛开展群众性精神文明创建活动，切实推进全国文明城市创建工作。

　　各位代表，市政府决定今年继续围绕人民群众关心的热点难点问题，办好以下21件实事：一是努力缓解人民群众看病难、看病贵，完成40所乡镇卫生院标准化建设工程，新型农村合作医疗农民参合率达到85％以上，在各县（市、区）全面推行城市医疗救助制度，医院药品价格平均下降30％左右；二是改善农村办学条件，完成100所农村中小学远程教育示范校建设，提升100所农村寄宿制学校建设和管理水平，支持30所农村寄宿制小学配置暖气；三是改善市区办学条件，抓好晋城二中、中原街中学、凤台中学、晋城三中、晋城九中、晋城十中、凤鸣小学、星河学校和秀水苑学校新建扩建项目；四是开工建设晋城学院；五是完善企业职工养老和工伤保险，提高企业退休人员基本养老金标准，基本实现所有煤矿、非煤矿山企业包括农民工在内的职工参加工伤保险；六是改善城镇困难居民住房状况，建设安康居住工程37.46万平方米，为140户特困居民解决廉租住房；七是继续实施国有重点煤矿棚户区改造和采煤沉陷区治理，国有重点煤矿建成住宅510套，采煤沉陷区治理建成住宅980套、货币补偿3360户；八是实施平安晋城“天网工程”，建设市区社会治安视频监控系统；九是实施城市“畅通工程”，重点完善景西路，建设前进路和市区东南出入口通道工程；十是实施市区煤层气综合开发利用项目，年内具备供气条件；十一是继续推进市区集中供热，新增供热能力180万平方米；十二是改善生态环境，市区空气质量二级以上天数达到295天以上，二氧化硫排放总量控制在11.33万吨、化学需氧量排放总量控制在1.7万吨，集中式饮用水水源地水质达标率达到100％；十三是实施六大造林生态绿化工程，重点抓好一二级国省道绿化、宽林带景观通道绿化、交通沿线荒山绿化和环城绿化；十四是实施生态家园富民工程，农村清洁能源用户达到10万户；十五是改善农村生产生活条件，解决10万农村人口饮水安全问题，修建通村水泥（油）路600公里，具备条件的建制村全部通公路；十六是治理农村地质灾害，对采矿主体灭失地因采矿造成村庄塌陷、房屋损坏的15个地质灾害村进行集中治理；十七是帮助农民就业和脱贫致富，培训农民10万人，转移农村富余劳动力1万人，搬迁移民50人以下的自然村100个；十八是继续实施“万村千乡市场工程”，发展连锁农家店260家；十九是实施“双千双百惠农工程”，支持“三农”贷款80亿元以上，为农民提供信贷服务25万户以上，支持90个带动农民增收的龙头企业，扶持25个农民专业合作社；二十是关心帮助农村特困群众生活，农村低保对象年人均补助360元，农村五保对象按标施保；二十一是继续扶持畜牧业发展，资助规模养殖和园区养殖，确保农民人均畜牧业纯收入增长8％以上。

　　各位代表，繁重而艰巨的工作任务，要求我们必须进一步加强政府自身建设。一是要进一步转变政府职能。完善权责明确、行为规范、监督有力、协调高效的行政管理体制，强化政府的经济调节、市场监管、社会管理和公共服务职能，坚决把政府不该管、也管不好的事交给市场、企业、社会组织和中介机构，保证各级政府机关及其工作人员严格按照法定权限和程序履行职责。继续深化行政审批制度改革，进一步减少审批事项，简化审批程序。着力完善和规范决策程序，提高政府决策的科学化、民主化水平。二是要进一步改进工作作风。大兴调查研究之风，深入基层，深入实际，了解民意，集中民智，理清工作思路，把握工作重点，提出有针对性的工作措施。大兴求真务实之风，坚持重实际、办实事、出实招、求实效，切实减少“文山会海”和不必要的应酬，取消不必要的检查、评比和验收活动，集中精力狠抓工作落实。大兴协调配合之风，各级各部门要紧紧围绕又好又快发展的总体要求，找准坐标，摆正位置，雷厉风行，狠抓落实，不推诿扯皮，不推三阻四，确保政令畅通，令行禁止。三是要进一步提高行政效率。完善工作目标责任制，任务到事，责任到人，加强督查，严格考核。坚决克服办事拖拉、敷衍塞责的官僚主义习气，坚决克服部门之间存在的“软摩擦”，坚决克服公务员队伍中存在的“懒惰性”，从自身做起，从工作的每个环节做起，强化效能建设，提高行政效率。严肃查处各种行政不作为、乱作为行为，优化政务环境，提高政府公信力和执行力。四是要进一步加强廉政建设。建立健全教育、制度、监督并重的惩防体系，全面落实政府系统党风廉政建设责任制，加强行政监察和审计监督，坚决纠正损害群众利益的各种不正之风，树立勤政、为民、廉洁的政府形象。政府系统各级领导干部和广大公务员要认真贯彻中央关于廉洁自律的各项规定，切实增强反腐倡廉意识，努力养成八个方面的良好风气，常修为政之德、常思贪欲之害、常怀律己之心，在廉洁自律方面发挥好带头作用。

　　各位代表，我们面临的任务光荣而艰巨，我们肩负的责任神圣而重大。让我们高举邓小平理论和“三个代表”重要思想伟大旗帜，在中共晋城市委的正确领导下，坚持以科学发展观和构建社会主义和谐社会战略思想统领经济社会发展全局，振奋精神，真抓实干，锐意进取，勇争一流，全力推进全市经济社会又好又快发展，为把我市建设成为经济发达、人民富裕、生态良好、社会和谐的新兴现代化城市而努力奋斗！
